# Animal Toxins Can Alter the Function of Nav1.8 and Nav1.9

**DOI:** 10.3390/toxins4080620

**Published:** 2012-08-14

**Authors:** John Gilchrist, Frank Bosmans

**Affiliations:** 1 Department of Physiology, School of Medicine, Johns Hopkins University, Baltimore, MD 21205, USA; Email: john.gilchrist@jhmi.edu; 2 Solomon H. Snyder Department of Neuroscience, School of Medicine, Johns Hopkins University, Baltimore, MD 21205, USA

**Keywords:** Nav1.8, Nav1.9, pain, animal toxins, voltage sensor, voltage-activated sodium channel

## Abstract

Human voltage-activated sodium (Nav) channels are adept at rapidly transmitting electrical signals across long distances in various excitable tissues. As such, they are amongst the most widely targeted ion channels by drugs and animal toxins. Of the nine isoforms, Nav1.8 and Nav1.9 are preferentially expressed in DRG neurons where they are thought to play an important role in pain signaling. Although the functional properties of Nav1.8 have been relatively well characterized, difficulties with expressing Nav1.9 in established heterologous systems limit our understanding of the gating properties and toxin pharmacology of this particular isoform. This review summarizes our current knowledge of the role of Nav1.8 and Nav1.9 in pain perception and elaborates on the approaches used to identify molecules capable of influencing their function.

## 1. Introduction

Of the nine voltage-activated sodium (Nav) channel isoforms found in humans (Nav1.1–1.9), Nav1.9 remains the least understood [1]. More than a decade ago, the slowly activating and inactivating ionic currents generated by Nav1.9 were discovered in dorsal root ganglia (DRG) neurons [2] where it appears alongside the similarly slow currents produced by Nav1.8 [3,4,5,6]. Subsequent experiments suggest that these two Nav channel isoforms may also be expressed in tissues such as enteric neurons [7,8], heart [9], retina [10], dental pulp [11], and trigeminal ganglia [11,12]; however, the strategic presence of Nav1.8 and Nav1.9 in nociceptive DRG neurons inspired various genetic approaches geared towards exploring their role in pain signaling [6,13,14,15,16,17,18,19]. While such studies are valuable for revealing the physiological role of Nav channels, these methods are of limited use for discovering pharmacological tools that evoke specific responses from these channels. An approach that has been successfully employed to investigate the functional properties of ion channels is to express them in heterologous systems and record ionic currents using electrophysiological techniques [20,21,22,23]. Although Nav1.8 can be functionally expressed in *Xenopus* oocytes or mammalian cell lines [24,25] (albeit not abundantly), fundamental questions about the function and pharmacological sensitivities of Nav1.9 remain unanswered because previous attempts to express this channel in heterologous systems have been unsubstantiated [15]. In addition, studying Nav1.9-mediated currents in native DRG neurons is technically challenging because only a fraction of isolated neurons produce a measurable amount [24,25] and other Nav channel isoforms, such as Nav1.8, interfere with these measurements since they activate over a similar voltage range [26,27]. Despite the existing Nav1.9 expression difficulties, creative approaches have generated insights into its functional properties and revealed molecules that interfere with its gating mechanism. This review will highlight these approaches as well as the compounds found to influence Nav1.8 and Nav1.9.

## 2. The Role of Nav1.8 and Nav1.9 in Pain

Given the abundant expression of Nav1.8 and Nav1.9 in sensory neurons, multiple studies with genetically altered mice have provided important insights into the physiological roles of these Nav channel isoforms in pain perception [6,13,14,15,16,17,18]. (Nav1.3 and Nav1.7 are also thought to be involved in nociception [16] but fall beyond the scope of this review.) Knockout mice [13,17,28], as well as siRNA and antisense deoxynucleotide studies [29] suggest a contribution of Nav1.8 to inflammatory pain, neuropathic pain and response to noxious stimuli [13,28,30,31] whereas Nav1.9 knockout mice have a largely absent inflammatory hyperalgesia in response to inflammatory mediators [6,14,17,32]. In addition, behavioral assays on these mice implicate a role for Nav1.9 in the development of visceral mechanical hypersensitivity associated with acute inflammation [33]. Although Nav1.8 was reported to be critical for the perception of cold pain [18], it was recently shown that Nav1.9 also has a crucial task in the pathogenesis of neuropathic pain, and specifically in the development of cold, but not mechanical allodynia [17]. Bearing in mind the potential limitations of the various models used, contradictory results were obtained by intraplantar carrageenan injection tests which revealed a reduced inflammatory-induced mechanical hypersensitivity in Nav1.9^-/-^ mice [34].

**Figure 1:**
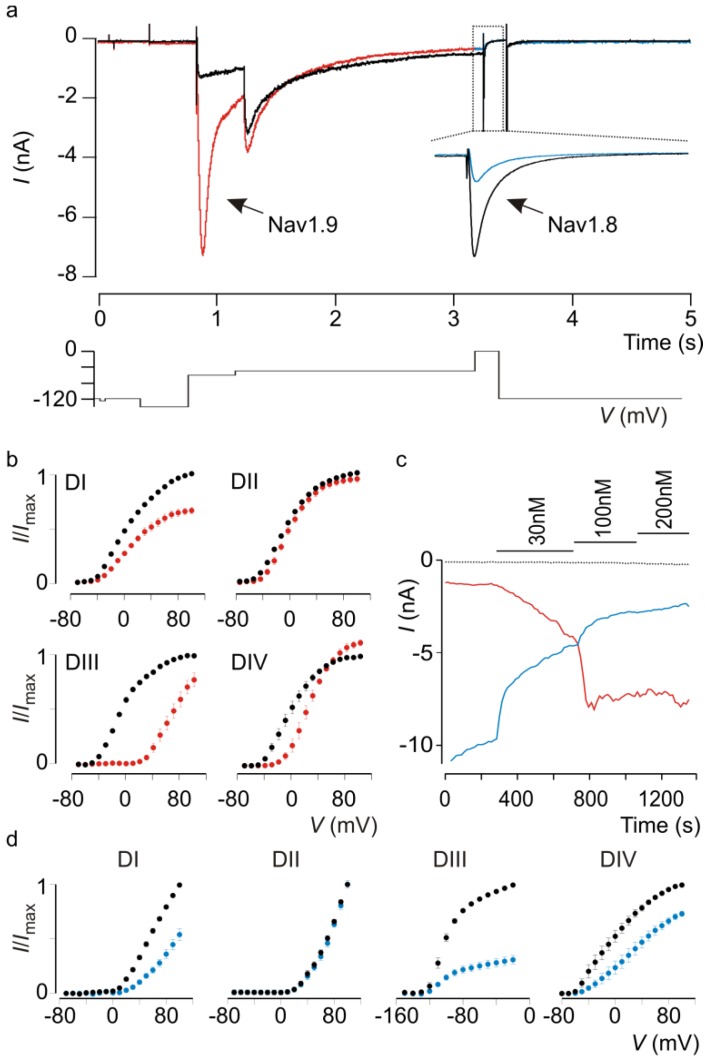
The tarantula toxin ProTx-I influences Nav1.8 and Nav1.9 currents in rat DRG neurons.

Detailed electrophysiological measurements on isolated sensory neurons suggest that Nav1.9 is unique in that it underlies the persistent sodium current in small diameter DRG neurons [26,27] (Figure 1a) that may drive spontaneous discharge during inflammation and that as such, unique DRG neuron properties such as subthreshold electrogenesis or oscillatory bursting discharges are absent in Nav1.9 knockout mice [3]. In addition, it was demonstrated that inflammatory mediators can dynamically regulate putative Nav1.9 currents in wild-type DRG neurons isolated from mice [3,5,6]. It is this apparent critical role in pain sensitivity that makes Nav1.8 and Nav1.9 desirable drug targets. Therefore, the discovery of molecules capable of modulating the slow currents of these particular Nav channel isoforms will be of great value to pharmacologically dissect their physiological role in wild-type DRG neurons. To this end, challenges associated with identifying and recording Nav1.8 and Nav1.9 currents must be addressed. 

## 3. Current Approaches for Studying Nav1.8 and Nav1.9 Function

One way to investigate the underlying molecular mechanisms that govern Nav channel gating is to remove the channel from its native environment, express it in heterologous systems such as *Xenopus* oocytes or mammalian cells, and record its ionic current in isolation. With varying degrees of success, this approach has been effectively employed for almost all Nav channel isoforms, yet functional expression of Nav1.9 remains a challenge [15,35,36]. Although successful recordings of Nav1.9 ionic currents in a mammalian cell line have been reported [37], the results have yet to be substantiated. Challenges of a different sort arise when attempting to measure Nav1.9-mediated currents in native tissues [4]. For example, Nav1.9 expression varies greatly between different types of DRG neurons [14,38], with most successful recordings originating from small-diameter (≤30 µm) capsaicin-sensitive neurons [39] (Figure 1a). Furthermore, the presence of ionic currents generated by Nav1.8 (Figure 1a) interferes with the identification of those produced by Nav1.9 as both isoforms are active over a similar membrane voltage range and selective inhibition of Nav1.8 with a pharmacological agent is difficult to accomplish [2,4,25,27]. To sidestep these technical limitations, various groups have come up with inventive solutions. For instance, the majority of Nav1.9 gating data was obtained by recordings from Nav1.8 knockout mice DRG neurons in which only the Na_v_1.9-mediated persistent current is present (in combination with exposure to tetrodotoxin to inhibit other Nav channel subtypes—see next section). Alternatively, researchers interested in exploring the functional properties of Nav1.9 in wild-type DRG neurons may add fluoride to the intracellular solution, thereby shifting the Nav1.9 gating characteristics to more hyperpolarized voltages [3,4,14,40]. Because Nav1.8 gating is essentially unaltered upon fluoride application, this method results in a narrow voltage window in which Nav1.9-generated sodium currents can be examined (Figure 1a). Although clever, this approach does require holding the membrane at voltages that are physiologically irrelevant. It is also possible to transfect the human (h)Nav1.9 clone into DRG neurons of a Nav1.9 knockout mouse, resulting in the restoration of certain pain sensations in addition to spontaneous firing patterns attributed to Nav1.9 function [5]. While promising, this approach suffers from the large volume of work required to produce, identify, and measure hNav1.9-expressing neurons.

However, as a result of all these efforts a body of work is now available that highlights the unique functional characteristics of Nav1.9. Compared to other Nav channel isoforms, Nav1.9-generated currents in DRG neurons reveal ultraslow activation and inactivation kinetics (Figure 1a) which is likely the product of a substantially different amino acid sequence, particularly in the voltage-sensing regions [25,41]. In addition, activation and steady-state inactivation relationships display significant overlap, suggesting a role for Nav1.9 in establishing the membrane voltage which may in turn influence gating of other Nav channel isoforms present in DRG neurons and thus, overall neuronal excitability [2]. As a result of its slow gating characteristics, it is generally agreed that Nav1.9 is not directly responsible for action potential generation. A potential mechanism contributing to the unique Nav1.9 gating process was reported recently when an established protein engineering approach was adapted to circumvent heterologous expression obstacles [25]. By identifying and transplanting structural motifs from the putative voltage sensor in each of the four Nav1.9 domains (I–IV) into the corresponding position in four-fold symmetric voltage-activated potassium (Kv) channels (Figure 1b), it became possible to investigate the individual contribution of these motifs to channel gating. One of the most intriguing results of this study is that Kv channels containing defined Nav1.9 voltage-sensing regions from domains II, III, and IV exhibit slow kinetics comparable to those observed for domain IV from Nav1.2 and Nav1.4. This suggests that the distinct gating characteristics of Nav1.9 observed in DRG neurons may result from slow activation of its voltage sensors. Notwithstanding the importance of identifying structural motifs within Nav1.9 voltage sensors that modulate overall channel function, other regions undoubtedly influence channel function as well. Therefore, heterologous expression of the full-length channel remains highly desirable. Nonetheless, a chimeric approach may be a valuable tool to discover molecules such as animal toxins that can influence Nav1.9 gating through an interaction with one or more voltage sensors.

Effect of ProTx-I on Nav1.9 and Nav1.8 currents in rat DRG neurons and chimeric ratNav1.9/Kv2.1 constructs. (a) Evoked currents in control conditions (black) and after addition of 100 nM ProTx-I (red for Nav1.9, blue for Nav1.8). Nav1.9 currents are greatly potentiated whereas Nav1.8 currents are strongly inhibited. Time scale for Nav1.9 currents is indicated in the X-axis. Voltage protocol used to elicit both Nav1.9 (first depolarization to −55 mV) and Nav1.8 currents (second depolarization to 0 mV) in a rat DRG neuron is shown below the trace. For clarity purposes, insert showing Nav1.8 inhibition by ProTx-I was obtained at 0mV from a DRG neuron held at −50 mV until Nav1.9 currents were fully inactivated. (Experiments performed by Michelino Puopolo, Stony Brook Medicine). (b) Effect of ProTx-I on chimeric constructs where paddle motifs were transferred from ratNav1.9 into ratKv2.1. Normalized tail current voltage-activation relationships are shown where tail current amplitude is plotted against test voltage before (black) and in the presence of toxin (red). Concentration used is 100 nM. The holding voltage was −90 mV, the test pulse duration was 300 ms, and the tail voltage was −60 mV. *n* = 3, and error bars represent SEM. (c) Concentration-dependent effect of ProTx-I on Nav1.9 currents (red) at −55 mV and Nav1.8 currents (blue) at 0 mV recorded in DRG neurons. Dotted trace represents leak current measured at −120 mV. Time scale is indicated on the X-axis. (d) Effect of ProTx-I on chimeric constructs where paddle motifs were transferred from ratNav1.8 into ratKv2.1. Normalized tail current voltage-activation relationships are shown where tail current amplitude is plotted against test voltage before (black) and in the presence of toxin (blue). Concentration used is 100 nM. *n* = 4, and error bars represent SEM.

## 4. Pharmacological Properties of Nav1.8 and Nav1.9

Nav1.8 channels interact with various commercially available compounds such as menthol [42], lidocaine [43], tetracaine [44], vinpocetine [45], ambroxol [46], lamotrigine [44], mexilitine [47], veratridine [48], and A-803467 [49], whereas very few animal toxins have been shown to be capable of reshaping Nav1.8 currents. ProTx-I and ProTx-II from the *Thrixopelma pruriens* tarantula were among the first spider toxins to be characterized as potent inhibitors of Nav1.8 opening [50], possibly by stabilizing one or more of its voltage sensors in the closed state [25,51,52] (Figure 1a,c,d). In 2006, CcoTx1, CcoTx2, and CcoTx3 from the *Ceratogyrus cornuatus* tarantula and PaurTx3 from the *Phrixotrichus auratus* tarantula were reported to inhibit rat Nav1.8 currents, albeit at high concentrations [53]. Although other spider toxins such as JZTX-V [54] and JZTX-IX [55] from the Chinese spider *Chilobrachys jingzhao* may also inhibit Nav1.8, their potential inhibitory effects have yet to be confirmed in heterologous systems. The µO-conotoxin MrVIB is a well-characterized Nav1.8 blocker isolated from the venom of the cone snail *Conus marmoreus* and is capable of drastically reducing Nav1.8-generated currents at nanomolar concentrations [56,57]. Although the toxin does not selectively target Nav1.8, intrathecal infusion of MrVIB in rat inflammatory pain models reduces allodynia and hyperalgesia [57]. Mutagenesis experiments on various Nav channel isoforms suggest that MrVIB exerts its effect by associating with pore loops in domain II and domain III within Nav1.8 and Nav1.4, respectively [58,59]. Finally, Nav1.8 function is profoundly modulated by batrachotoxin, an extremely potent toxin found in the skin secretions of poison dart frogs and certain birds endemic to New Guinea [60]. Mechanistic studies on several Nav channel variants suggest that batrachotoxin may interact with residues within the pore or at the channel-lipid interface to cause persistent channel activation [61,62]. Although most of the aforementioned toxins could prove to be promising lead compounds for drug design, one caveat is that their efficacy on the hNav1.8 variant remains to be determined.

Compared to other Nav channel isoforms, the discovery of ligands capable of influencing Nav1.9 function has been hindered by the absence of a reliable heterologous expression system. However, studies on wild-type Nav1.9 channels expressed in neuronal tissues from various animals have revealed a variety of endogenous molecules that can influence channel function. Pertinent to its role in pain perception, inflammatory mediators such as PGE2 and serotonin [6,63] as well as secreted proteins that augment neuronal survival (e.g., brain-derived neurotrophic factor (BDNF) [35] and glial-derived neurotrophic factor (GDNF) [64,65]) have been reported to increase Nav1.9 currents in DRG neurons. While these compounds may individually influence Nav1.9 expression or through a G-protein coupled mechanism [6], a “soup” consisting of multiple inflammatory mediators such as bradykinin, histamine, PGE3 and norepinephrine can also act together to achieve a similar increase in Nav1.9-mediated currents [3]. Finally, the neurokinin-3 receptor agonist, senktide, was shown to potentiate Nav1.9 currents in guinea pig enteric afferent neurons [66], yet the mechanism underlying this observation or its translation to human DRG neurons has not been investigated. 

Although peptides isolated from venomous organisms have proven their value in probing the role of ion channels in neuronal excitability and to gain insight into their intricate gating machinery [67], the exploration of Nav1.9 toxin sensitivity is still in its infancy. Tetrodotoxin (TTX) and saxitoxin (STX) are naturally occurring guanidinium toxins that interact potently with the Nav channel pore region and occlude the sodium ion permeation pathway [68]. TTX is widely used to divide the Nav channel family into two groups based on their sensitivity towards the toxin; TTX-sensitive channels (Nav1.1–Nav1.4, Nav1.6–Nav1.7) are inhibited by nanomolar concentrations whereas Nav1.8 and Nav1.9 require millimolar amounts to be blocked completely (Nav1.5 requires micromolar concentrations to be blocked). Interestingly, it was shown on one occasion that nanomolar concentrations of STX can reversibly block BDNF-evoked TTX-resistant sodium currents in CA1 pyramidal neurons and SH-SY5Y neuroblastoma cells [35], suggesting that Nav1.9 may be sensitive to this toxin. However, similar STX concentrations did not inhibit TTX-resistant currents in nociceptive neurons [6]. µ-conotoxins, a subset of conotoxins isolated from cone snail venoms, have been shown to compete with TTX and STX and inhibit ion flow through Nav channels by interacting with the pore region [69]. Interestingly, two groups simultaneously reported that the µ-conotoxin µ-SIIIA from *Conus striatus* selectively inhibits TTX-resistant but not TTX-sensitive currents in rat DRG neurons [70,71]. Although the authors suggest that this toxin and relatives such as µ-KIIIA [70] and µ-SmIIIAs [72] may primarily inhibit Nav1.8, a potential inhibitory effect of these peptides on Nav1.9 opening should not be ruled out. 

In recent work, chimeric constructs between Nav1.9 and various Kv channels were exploited to investigate the contribution of specific regions within Nav1.9 voltage sensors, the S3b-S4 paddle motifs, to forming animal toxin receptors [25]. These paddle motifs were originally identified in Kv channels in which their role in driving voltage sensor activation and subsequent opening of the pore makes them a prime target for tarantula toxins [73,74,75,76,77,78,79], possibly through a lipid-mediated mechanism [80,81,82]. Later, it was shown that paddle motifs are also an integral part of the voltage sensors in Nav1.2 and Nav1.4 where they are extensively targeted by a variety of toxin families [51]. Since the pharmacological sensitivities of Nav1.9 remain largely unexplored, chimeric constructs were employed to search for toxins that might interact with Nav1.9 channels (Figure 1b). To this end, eighteen toxins from tarantula, scorpion and sea anemone venom were screened against four Nav1.9/Kv2.1 paddle constructs. The two most interesting toxins that emerged were the scorpion toxin TsVII from *Tityus* *serrulatus* and the tarantula toxin ProTx-I, both of which interact strongly with Nav1.9 paddle motifs (Figure 1b) and potently facilitate the slowly activating and inactivating Nav1.9-mediated current in rat DRG neurons (Figure 1a). In addition to targeting Nav1.9, TsVII and ProTx-I have very different actions on Nav1.8; TsVII produces a concentration-dependent facilitation of Nav1.9 currents while only marginally inhibiting Nav1.8, whereas ProTx-I causes both a pronounced potentiation of Nav1.9 currents and a robust concentration-dependent inhibition of Nav1.8, thereby indicating that this toxin may be a formidable tool to discriminate between sodium currents generated by these two channel isoforms in DRG neurons (Figure 1a,c). Of interest is that both toxins possess a different paddle target profile when comparing the rat and human isoform. In addition, ProTx-I targets different voltage sensors in Nav1.2, suggesting that Nav1.9 has its own unique pharmacological profile that may be advantageous for drug design. 

## 5. Future Challenges

Although scientists have made astonishing progress in understanding the mechanisms and various types of pain, many gaps still remain and developing more effective pain relievers continues to be a major challenge. In particular, the enigmatic Nav channel Nav1.9 constitutes one of the most elusive pain targets, mainly due to various complications associated with identifying its sodium current in native neurons and the lack of reliable heterologous expression systems. In contrast, Nav1.8 can be expressed in *Xenopus* oocytes and mammalian cell lines; however, it can be challenging to obtain sodium current densities that are practical for conducting experiments. Moreover, Nav1.8 seems to be quite insensitive to animal toxins capable of inhibiting other Nav channel isoforms at low concentrations, possibly resulting from its unique amino acid composition of the paddle region. As a result, the search for and characterization of pharmacological agents that influence Nav1.8 and Nav1.9 action is severely lagging when compared to other Nav channel isoforms. However, various creative approaches have led to the discovery of endogenous molecules and animal toxins capable of altering the function of Nav1.8 and Nav1.9. With the advent of novel approaches to heterologous expression problems and high-throughput screening, it may be a matter of time before these intriguing Nav channels become readily available for detailed mechanistic studies of their unique gating properties and for screening animal venoms from which toxins that reshape channel activity can be isolated.
